# Benefits of a microprocessor-controlled prosthetic foot for ascending and descending slopes

**DOI:** 10.1186/s12984-022-00983-y

**Published:** 2022-01-28

**Authors:** Michael Ernst, Björn Altenburg, Thomas Schmalz, Andreas Kannenberg, Malte Bellmann

**Affiliations:** 1 Research Biomechanics, CR&S, Ottobock SE & Co. KGaA, Göttingen, Germany; 2 Medical Affairs, Otto Bock Healthcare LP, Austin, USA

**Keywords:** Prosthetics, Ramp walking, Microprocessor-controlled prosthetic feet, Prosthetic knee, Biomechanics

## Abstract

**Background:**

Prosthetic feet are prescribed for persons with a lower-limb amputation to restore lost mobility. However, due to limited adaptability of their ankles and springs, situations like walking on slopes or uneven ground remain challenging. This study investigated to what extent a microprocessor-controlled prosthetic foot (MPF) facilitates walking on slopes.

**Methods:**

Seven persons each with a unilateral transtibial amputation (TTA) and unilateral transfemoral amputation (TFA) as well as ten able-bodied subjects participated. Participants were studied while using a MPF and their prescribed standard feet with fixed ankle attachments. The study investigated ascending and descending a 10° slope. Kinematic and kinetic data were recorded with a motion capture system. Biomechanical parameters, in particular leg joint angles, shank orientation and external joint moments of the prosthetics side were calculated.

**Results:**

Prosthetic feet- and subject group-dependent joint angle and moment characteristics were observed for both situations. The MPF showed a larger and situation-dependent ankle range of motion compared to the standard feet. Furthermore, it remained in a dorsiflexed position during swing. While ascending, the MPF adapted the dorsiflexion moment and reduced the knee extension moment. At vertical shank orientation, it reduced the knee extension moment by 26% for TFA and 49% for TTA compared to the standard feet. For descending, differences between feet in the biomechanical knee characteristics were found for the TTA group, but not for the TFA group. At the vertical shank angle during slope descent, TTA demonstrated a behavior of the ankle moment similar to able-bodied controls when using the MPF.

**Conclusions:**

The studied MPF facilitated walking on slopes by adapting instantaneously to inclinations and, thus, easing the forward rotation of the leg over the prosthetic foot compared to standard feet with a fixed ankle attachment with amputation-level dependent effect sizes. It assumed a dorsiflexed ankle angle during swing, enabled a larger ankle range of motion and reduced the moments acting on the residual knee of TTA compared to the prescribed prosthetic standard feet. For individuals with TFA, the prosthetic knee joint seems to play a more crucial role for walking on ramps than the foot.

## Introduction

A lower-limb amputation, regardless of the anatomical level, is a dramatic event that inevitably results in mobility impairments. Prostheses are designed to help restore at least part of the lost mobility. Nonetheless, walking on ramps or walking on uneven ground as part of independent community ambulation are still challenging tasks for these persons, especially with the current standard of care of energy-storage-and-return (ESR) feet with a fixed ankle attachment.

ESR feet show benefits compared to conventional prosthetic feet, such as solid-ankle cushioned-heel feet, by enabling their users to walk with a nearly natural motion pattern over a large range of speeds on level ground [1–3]. However, as standard ESR feet provide no movement in the ankle and can adapt to non-level surfaces like slopes, stairs or uneven ground only by the flexibility of their springs, these situations are challenging to master for individuals with lower-limb amputations. For example, a study investigating standing on slopes found that persons with a transtibial amputation (TTA) and persons with a transfemoral amputation (TFA) had to use postural compensation strategies to cope with the lack of adaptability in ESR prosthetic feet [4]. Different design concepts have been implemented in commercially available feet that are thought to facilitate walking on slopes. For ESR feet, it was shown that a linkage joint system may increase the range of motion (ROM) of the ankle and simultaneously enhance energy return [5, 6]. A similar effect on the ROM might be achieved with decreasing the stiffness of the springs of ESR feet, but that could also negatively affect the energy efficiency and the roll-over behavior of the feet on level ground [7]. Another concept is to actively control an integrated ankle joint by adapting hydraulic plantar- and dorsiflexion resistances to the terrain to control the ROM [8–10] and regulate shank rotation velocity [11]. Such an adaptation likely shifts the foot’s neutral point—the angular position at which the acting external plantar- and dorsiflexion moments cancel each other out [12]. Another terrain adaptation method may be realized with a motor that automatically adapts the angle of an integrated ankle joint during swing phase [13, 14]. It adapts the ROM of the entire foot and it is plausible that such adaptation also shifts the foot’s neutral point.

Microprocessor-controlled prosthetic feet (MPF) may have the potential to improve slope and uneven terrain ambulation in individuals with TTA and TFA. Studies have found that persons with TTA and TFA may benefit in slope ambulation from prosthetic foot features like a larger ROM of the ankle [5, 9, 15, 16], active ankle control [9, 11, 13, 16–18], better energy storage and return [5] or powered ankle support [19, 20]. Such benefits were characterized by decreased residual and sound side loading of muscles and joints, reduced pressure peaks in the socket-residual limb interface, reduced metabolic energy consumption, increased perceived comfort and safety, and minimized compensatory movements during ambulation on slopes. With focusing on commercially available MPF without powered ankle support, previous studies on slope ambulation have shown mixed results with improvements in some biomechanical parameters but deterioration in others [21], further complicated by high inter-subject variability [13]. Many of these studies were limited by the only gradual and partial adaptation of the studied MPF to slopes [13, 21] or testing on only shallow slopes of 5° of a MPF with very limited ROM at the ankle joint [11, 18, 22]. Moreover, the vast majority of studies investigated slope ambulation with MPF only for persons with TTA.

The aim of this study was to investigate how a current hydraulic MPF adapts to steeper slopes and what effects it has on the gait of individuals with lower-limb amputations compared to their prescribed ESR feet with rigid ankles attachments. It was hypothesized that the MPF adapts better to inclinations and, thus, facilitates walking on slopes for its user. These adaptations might change the forward rotational resistance behavior of the leg over the prosthetic foot and the MPF’s neutral point. Another research question was whether the adaptation process showed the same characteristics in subjects with different lower-limb amputation levels. To answer these questions, kinematic and kinetic data of the prosthetic side were measured for the knee and ankle joints of individuals with TTA and TFA as well as able-bodied controls while ascending and descending a slope of 10°.

## Methods

Seven persons with a unilateral TTA and seven subjects with a unilateral TFA participated in the study. Demographic and prosthetic componentry details are presented in Table 1. All subjects were physically active in their daily lives with mobility levels MFCL-3 and 4 and used ESR feet. Furthermore, individuals with TFA used microprocessor-controlled knee joints (MPK) in their daily lives. As controls, 10 able-bodied subjects underwent the same gait tests to obtain reference data.

**Table 1:** Subject demographics and prostheses used in daily live

Subject	Level of amputation	Age in years	Weight in kg	Height in cm	Years since amputation	Stump length^a^	Reason for amputation	Prostheses—feet and knees
P#1	TT	48	68	183	3	Short	Infection	Trias 1C30
P#2	TT	74	84	174	15	Medium	Aterial occlusion	C-Walk 1C40
P#3	TT	48	80	177	13	Medium	Trauma	Triton Harmony 1C62
P#4	TT	56	87	178	35	Medium	Trauma	C-Walk 1C40
P#5	TT	39	94	168	7	Medium	Trauma	Triton 1C60
P#6	TT	48	81	181	38	Medium	Trauma	Triton 1C60
P#7	TT	49	77	168	32	Medium	Cancer	Triton LP 1C63
P#8	TF	32	81	184	14	Medium	Trauma	Triton 1C60 & X3
P#9	TF	52	85	177	26	Medium	Trauma	Triton 1C60 & X3
P#10	TF	41	91	182	28	Medium	Trauma	Triton 1C60 & X3
P#11	TF	44	75	169	39	Medium	Trauma	Triton 1C60 & Genium
P#12	TF	48	76	178	25	Medium	Trauma	Triton 1C60 & C-Leg 3
P#13	TF	45	83	184	22	Medium	Trauma	C-Walk 1C40 & C-Leg 3
P#14	TF	61	105	186	39	Long	Trauma	Triton 1C60 & Genium
Mean TT		52 ± 10	82 ± 8	176 ± 5				
Mean TF		46 ± 7	85 ± 10	180 ± 5				
Controls		23 ± 3	71 ± 13	173 ± 8				

^a^Definitions residual limb length: short < 1/3, medium 1/3—2/3, Long > 2/3 length of sound side limb segment; anatomical landmarks for measurements: TF Tuber ischium—residual limb end | TT Medial Tibial Plateau—residual limb end

The study was approved by the Ethics Committee of the University Medical Center Göttingen (UMG), Germany, and conducted in accordance with the Declaration of Helsinki. Prior to the study, all subjects received a detailed introduction in the planned testing procedure and signed a written informed consent.

### Prosthetic foot

In this study, the participants´ prescribed ESR feet with rigid ankle attachments and the microprocessor-controlled prosthetic foot Meridium (Otto Bock, Germany; further referred to as MPF-M) were used. All subjects with TFA were fitted with MPK of the same type (Genium, Otto Bock, Germany) for the duration of the study to minimize the variability between the prosthetic knee joints. An optimized alignment process of the prosthesis as recommended by the manufacturer (static, dynamic, MPF-M specific settings) was ensured by a certified prosthetist. The participants received a dedicated training (walking on level ground, stairs, uneven terrain; turning, stopping, sitting, etc.) supervised by the prosthetist to accommodate to the foot and its functionality in a first step. Before the tests with the MPF-M were conducted, the participants were also given a home-use accommodation period of at least one week. Participants performed all tests with their prescribed feet in the first visit, and with the MPF-M after the accommodation period in a second visit. The test situation “Ramp Walking” was trained with each foot prior to the respective measurement session to familiarize the participants with the test setup.

**Fig. 1:**
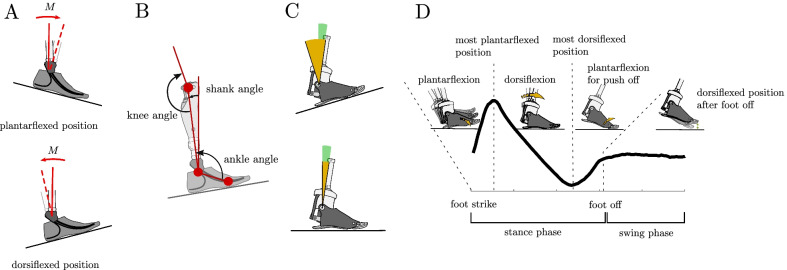
Schematic illustration of the acting ankle moments and ankle motion on a slope. **A** Acting internal moments at the ankle due to the foot´s deflection, **B** sagittal angles on the slope, **C** ankle angle (yellow) and the maximum dorsiflexion angle (green) for the MPF-M and **D** schematic illustration of the MPF-M ankle motion for one gait cycle. **A** If the shank is rotated to an upright position from its neutral point (torque free position—dashed red line), the carbon heel spring is deflected and creates, due its internal moment M, a dorsiflexion moment for Down or a plantarflexion moment for Up, respectively. During Down, it pulls the knee into flexion and, during Up, it counteracts the forward rotation of the shank. Note that the reported external ankle moments act inversely to the internal ones. **B** Studied kinematic parameters were estimated for the sagittal plane—ankle angle (angle between toe, ankle and knee markers), knee angle (angle between ankle, knee, and trochanter markers) and shank angle (angle between ankle-knee marker line and vertical axis). (C) The MPF-M’s maximum dorsiflexion angle (green) is constant relative to the shank angle for level and UP. The ankle angle, in contrast, varies for the same shank angle

The studied MPF-M has a polycentric design (4 axis) and generates hydraulic plantar- and dorsiflexion resistances. The microprocessor utilizes sensor data from an inertial motion unit (gyroscopes and acceleration sensors), a sagittal ankle moment and an angle sensor. Sensor input to the microprocessor and control loops work with 100 Hz. The MPF-M offers additional functionality compared to ESR feet with rigid ankle attachments [4, 8]. The most important functions related to walking are an instantaneous adaptation to inclines and declines during stance of every single step. For that purpose, it detects the current tilt of the surface in early stance and adjusts plantarflexion and dorsiflexion hydraulic resistances and the maximum hydraulic dorsiflexion angle for the same step accordingly. With the example of an upslope condition, it will extend the possible maximum dorsiflexion by the amount of deviation of the surface tilt from level. Effectively, the hydraulic blocks further dorsiflexion beyond this shank angle for level and upslope walking (Fig. 1C). For that purpose and for adapting the rotational resistance the shank angle is used as an input parameter in the control loops. This characteristic distinguishes the MPF-M from standard ESR feet without an ankle joint. It can be assumed that in ESR feet with a rigid ankle attachment, the forward rotational resistance as a function of the shank angle depends on the tilt of the surface and the stiffness of the springs (Fig. 1A; Fig. 11 in [17]).

The MPF-M has a maximum plantarflexion of 22° and dorsiflexion of 14°. The MPF-M has a carbon heel and a titanium ankle spring. The push-off is realized by a toe plate that is mechanically coupled with the four-bar mechanism. The amount of push-off is influenced among other factors by the individual dorsiflexion damping settings for the user (for a comparison to other feet see [12]). The foot remains in a dorsiflexed position during swing phase for increased toe clearance. Furthermore, it has different settings for the user’s level of amputation (TTA or TFA).

The term “neutral point” is used for the angular position of the ankle at which the sum of external moments acting on the ankle in the stance phase is zero, see Fig. 1A. It is plausible that a change in plantarflexion and dorsiflexion hydraulic resistances may result in a shift of that point [12].

### Setup

Slope ascent (“Up”) and descent (“Down”) were measured on a ramp of 3 m length with a 10° inclination. For safety reasons, a handrail was attached to the ramp. A slope of 10°, which is moderate but already challenging for persons with a lower-limb amputation, was used to investigate adaptation effects. It is an inclination that is often used in gait experiments, e.g. [4, 9, 23–25]. A force plate (Kistler 9287A, 1000 Hz, Kistler Group, Switzerland) installed on the ramp was used to measure kinetic data of one gait cycle. Due to the ramp setup, we captured the first ground contact of the prosthetic side on the slope for Up. For Down, the second or third step was measured depending on person’s step length.

Seventeen markers were used to record kinematic data of the subjects and prostheses with 12 Vicon cameras (Vicon Bonita, 200 Hz, Vicon Motion Systems, UK). Motion tracking markers were placed bilaterally, among others on the toe part (equivalent position to first metatarsophalangeal joint), ankle joint (mechanical foot rotation axis for MPF-M, equivalent for ESR), knee joint, and greater trochanter, see Fig. 1B.

Prior to each measurement session, participants accommodated to the lab environment and test setup. At least seven valid trials with one gait cycle each were recorded for each situation (ramp Up/Down, MPF-M/ESR). The validity of the trials was determined by an assessor next to the track (inclusion criteria: constant walking speed, entire foot on the force plate without obviously aiming for it and without specific step length adaptation, no handrail use).

### Data processing

Kinematic and kinetic data were filtered (4^th^ order zero-lag Butterworth low pass filter with 15 Hz cut-off) and processed with customized Vicon BodyBuilder scripts (Vicon Motion Systems, UK) to calculate joint angles and external joint moments of the gait cycles (GC). Vertical and horizontal ground reaction forces (GRF), external sagittal ankle and knee moments, sagittal shank angle and sagittal joint angles of the prosthetic leg were calculated.

The joint angles were referenced to static-standing trials. The shank angles were calculated with respect to the vertical axis, see Fig. 1B. A shank angle of zero (SA = 0) indicates a vertical shank orientation. The shank angle is utilized for the control of the MPF-M, and the ankle moment as a function of the shank angle seems to be an invariant in human walking over uneven ground [17]. Therefore, ankle and knee moments were investigated with respect to the shank angle and analyzed for the specific angle of SA = 0. The ankle moment at SA = 0 is used as an indicator for the forward rotational resistance of the shank over the foot.

Ground reaction forces (GRF) and external moments in the sagittal plane were normalized to the body mass of the participants. The results for the groups of individuals with TTA and TFA, respectively, are reported separately to account for effects related to the level of amputation.

Individual means of the gait cycle parameters were calculated from the single trials, and group means were calculated from the individual means. Parameters were tested for differences between feet within the groups of subjects with TTA and TFA using a Wilcoxon signed-rank test. The alpha level for significance was set to 5%. Since we had reason to assume that the ankle angle values (ROM, maximum dorsiflexion and plantarflexion, angle in swing) with the MPF-M are larger than with the ESR (features of the MPF-M and studies [4, 9, 12]), we used one-sided hypotheses to test for differences. Furthermore, since the MPF-M adapts the forward rotational resistance, it was expected that the magnitude of the ankle moment at SA = 0 was reduced (one-sided hypotheses). For all other statistical tests, two-sides hypotheses were used. The effect size r was estimated for statistically significant differences using z-values of the Wilcoxon signed-rank test. Effects are considered large for |r|> 0.5 and moderate for |r|> 0.3, see [26, 27]. Differences between subject groups (TTA, TFA and controls) were not statistically tested. However, curves and parameter values were used for qualitative comparisons.

## Results

### Joint angle and moment characteristics for inclines

**Fig. 2:**
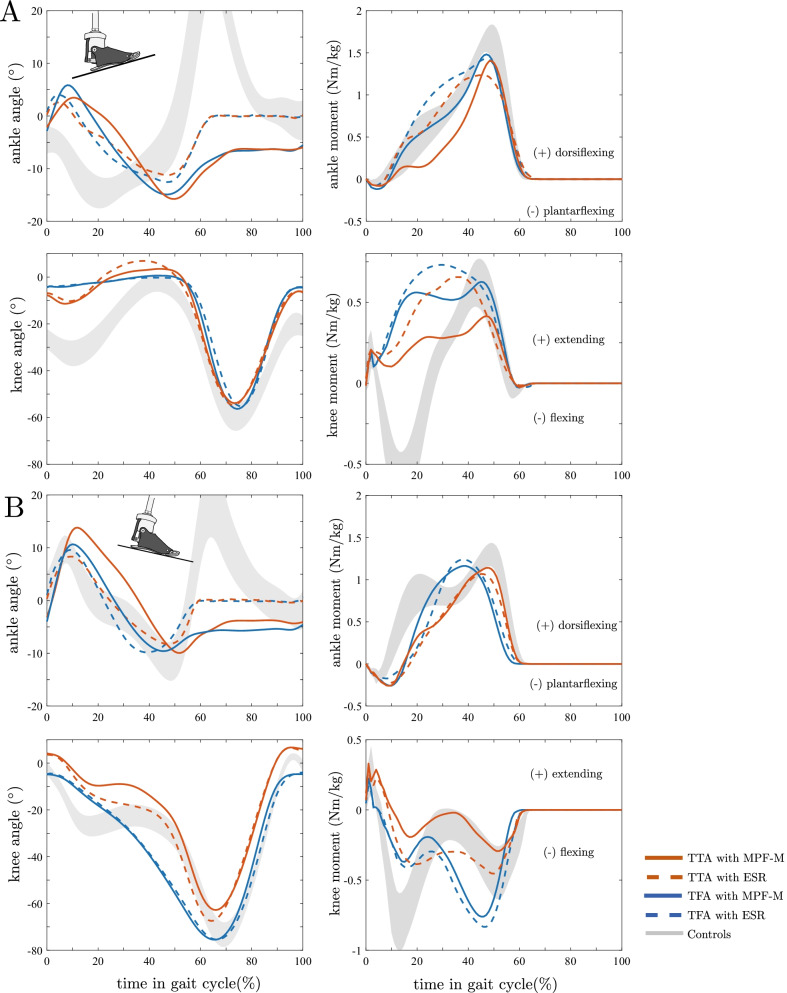
Group mean kinematics and kinetics for one gait cycle on the slope. Ankle angle, knee angle, ankle moment and knee moment characteristics (sagittal) for walking **A** Up and **B** Down the 10° slope. Curves: TTA (red), TFA (blue), and controls (grey area); ESR (dashed lines) and MPF-M (solid lines)

Joint angle characteristics for ascending the slope are presented in Fig. 2A and Table 2. Both feet showed an initial plantarflexion after heel strike followed by a dorsiflexion. Individuals with TTA and TFA walking with the MPF-M exhibited a larger plantarflexion (about 2°; TFA p < 0.01, r = 0.89; TTA p < 0.05, r = 0.64) and dorsiflexion (about 3°; TFA & TTA both p < 0.01, r = 0.89) compared to ESR, indicating a larger ankle ROM. Compared to the ESR feet, the mean ankle ROM increased from 14° to 20° in TTA (p < 0.02, r = 0.83) and from 17° to 22° in TFA (p < 0.01, r = 0.89) with the MPF-M. Furthermore, after push-off, the MPF-M remained in a dorsiflexed position of about 6° in TTA and 7° in TFA (both p < 0.02, r ≥ 0.83). In contrast, no initial plantarflexion motion but a dorsiflexion motion from 5° to 15°, followed by an earlier plantarflexion motion (at about 20% GC), was observed in the controls.

**Table 2:** Sagittal joint angles for the prosthetic side (TTA,TFA) and controls

	TFA		TTA		Controls
*Sagittal joint angles* (°)—*UP*	ESR	MPF-M	ESR	MPF-M	–
Ankle—most plantarflexed angle (early stance)	**4.2 ± 0.7**	**6.5 ± 2.0**	2.8 ± 2.0*	4.5 ± 1.9*	− 4.5 ± 2.5
Ankle—most dorsiflexed angle (mid stance)	− **12.7 ± 2.8**	− **15.3 ± 1.7**	− **11.5 ± 2.7**	− **15.9 ± 1.4**	− 14.8 ± 2.8
Ankle—range of motion	**17.0 ± 3.0**	**21.8 ± 2.5**	**14.3 ± 4.5**	**20.4 ± 1.2**	10.5 ± 2.5
Ankle—angle in swing	**0.1 ± 0.3**	− **6.5 ± 0.7**	− **0.1 ± 0.1**	− **6.4 ± 1.9**	− 1.5 ± 3.1
Knee—at foot strike	− 4.3 ± 1.0	− 4.4 ± 1.3	− 6.9 ± 9.0	− 7.7 ± 9	− 26.4 ± 4.3
Knee—most extended angle (in stance)	**0.3 ± 1.6**	**1.2 ± 0.6**	**7.6 ± 7.2**	**5.7 ± 4.5**	− 2.1 ± 3.8
Knee—most flexed angle (in swing)	− 56.7 ± 6.5	− 58.7 ± 4.9	− 55.5 ± 4.3	− 54.3 ± 5.7	− 60.0 ± 6.0
Shank—angle at foot strike	− **19.8 ± 2.8**	− **21.1 ± 2.8**	− 22.1 ± 5.0	− 21.9 ± 6.1	− 14.6 ± 3.1
Shank—angle standing (static trial)	6.1 ± 1.1	6.1 ± 1.1	6.3 ± 1.7	5.7 ± 2.2	3.9 ± 3.1
*Sagittal joint angles* (°)—*DOWN*					
Ankle—most plantarflexed angle (early stance)	9.9 ± 1.3	10.7 ± 2.9	**8.5 ± 3.8**	**14.4 ± 2.1**	9.7 ± 2.6
Ankle—most dorsiflexed angle (mid stance)	− 10.0 ± 2.6	− 9.9 ± 2.8	− 8.5 ± 1.9	− 10.4 ± 2.0	− 11.7 ± 3.6
Ankle—range of motion	19.8 ± 3.5	20.6 ± 3.3	**17.0 ± 5.6**	**24.8 ± 3.9**	21.6 ± 4.3
Ankle—angle in swing	− **0.2 ± 0.2**	− **5.6 ± 3.0**	**0.1 ± 0.1**	− **3.9 ± 2.0**	− 2.7 ± 3.1
Knee—at foot strike	− 4.8 ± 0.9	− 4.8 ± 1.2	3.6 ± 1.3	4.1 ± 2.8	− 2.3 ± 2.5
Knee—most flexed angle (in swing)	− 76.1 ± 4.9	− 76.2 ± 5.1	− **67.9 ± 3.3**	− **63.1 ± 5.6**	− 73.6 ± 5.3
Shank—angle at foot strike	− 9.8 ± 3.0	− 10.3 ± 2.1	− 14.0 ± 1.6	− 15.1 ± 3.7	− 12.7 ± 2.4
Shank—angle standing (static trial)	6.1 ± 1.1	6.1 ± 1.1	6.3 ± 1.7	5.7 ± 2.2	3.9 ± 3.1

Mean leg joint angles in ° ± SD shown. Statistical differences between the feet within a group (TTA, TFA) marked bold (p < 0.05). Values for controls are given for a qualitative comparison. Ankle angles in swing of controls are time dependent and are not constant. Values are differences to static trial, i.e. ankle angle: positive—more plantar flexed, negative—more dorsiflexed; knee and hip angle: positive—more extended, negative—more flexed

While differences in the sagittal knee angle characteristics were visible between the groups of TTA and TFA, Fig. 2A, only small variations were found between the feet. For Up, subjects with TFA showed an extended knee joint until terminal stance. No difference was observed between knee angles with the different feet. Subjects with TTA also walked with an almost extended knee joint but, in contrast to TFA, exhibited a flexion–extension motion of the knee with an effect of foot type on hyperextension (2° less with MPF-M, p < 0.05, r = 0.70), see Fig. 2A. Controls showed a more pronounced flexion–extension motion starting with a much more flexed knee at initial ground contact (about 26°).

For all subject groups and feet, an initial plantarflexion moment (at heel contact) followed by an increasing dorsiflexion moment (until 45 to 50% GC with 1.3 to 1.6 Nm/kg) was found, see Fig. 2A. TTA and TFA showed knee extension moments on the prosthetic side until swing initiation, see Fig. 2A. The characteristics of peak values and timing were dependent on foot type and amputation level. In contrast, the knee motion in the controls was accompanied by a flexion–extension sagittal knee moment characteristic.

**Fig. 3:**
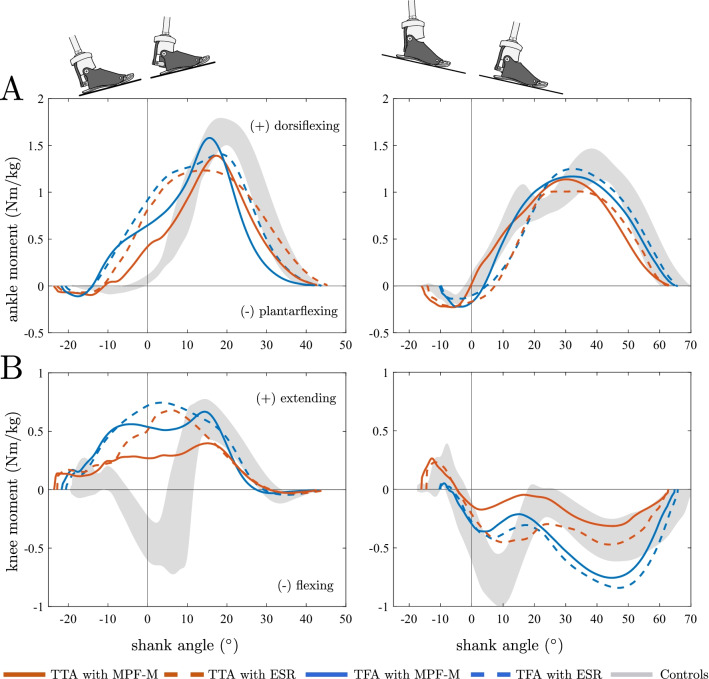
External ankle and knee moment as a function of the shank angle on the slope. **A** Ankle and **B** knee moment characteristics for walking Up (left column) and Down (right column) the 10° slope. A shank angle of 0° (SA = 0) indicates a vertically oriented lower leg. The neutral point is reached when the ankle moment curve crosses zero, which is approximately at a vertical shank angle in controls and situation-dependent in TTA and TFA. Curves: TTA (red), TFA (blue), and controls (grey area); ESR (dashed lines) and MPF-M (solid lines)

Figure 3 presents the external sagittal joint moments as a function of the shank angle. Shank angles did not differ significantly in the static standing trials between feet in the subject groups, see Table 2, and, thus, allowed to directly compare foot-dependent moments in relation to specific shank angles.

For UP, larger shank angles were observed in TTA and TFA compared to controls at initial ground contact, see Table 2. To subsequently reach an vertical shank position (from about − 21° to 0°), subjects had to overcome a dorsiflexion resistance. Differences between feet were observed for the ankle moment at vertical shank orientation (SA = 0°) with reductions of 29% and 49% with MPF-M compared to ESR in TFA (p < 0.02, r = 0.83) and TTA (p < 0.01, r = 0.89), respectively. In contrast, controls showed an almost moment-free ankle motion to the vertical shank position (from − 14.6° to 0°), see Fig. 3A. Furthermore, at that specific shank position, the knee extension moments observed in TFA and TTA were reduced by 26% and 49%, respectively, with MPF-M (both p < 0.03, r = 0.83). Controls showed knee flexion moments. The peak knee extension moments occurred at shank angles of about 5° with ESR and similar to those of the controls (about 15°) when using the MPF-M, see Fig. 3A.

### Joint Angle and Moment Characteristics for Declines

Joint angle characteristics for descending the slope are presented in Fig. 2B and Table 2. An initial plantarflexion after heel strike was observed for all groups and feet. The amount of plantarflexion and dorsiflexion differed between feet in TTA (p < 0.02, r = 0.83 and p < 0.05, r = 0.64) but not in TFA. The ankle ROM for TTA was about 25° with MPF-M (p < 0.03, r = 0.77) compared to 17° with ESR. For TFA, the ROM was about 20° with both feet, and 22° for controls. After push-off, the MPF-M remained in dorsiflexion of about 4° and 6° for TTA and TFA (both p < 0.01, r = 0.89), respectively.

A continuous knee flexion motion was found during stance in the TFA group which qualitatively did not differ between feet (yielding mode of Genium knee). The TTA group showed a foot-dependent knee flexion (p < 0.03, r = 0.83) with smaller values and a flexion–extension motion with the MPF-M. Controls used a similar, more pronounced knee flexion characteristic during stance phase.

Qualitatively compared to Up and controls, longer plantarflexion moments were acting in TTA and TFA with both feet in the initial 15% of GC. Furthermore, controls showed a double-hump characteristic that was not observed in TTA and TFA, see Fig. 2B ankle moment.

Knee flexion moments changed with amputation level and foot (only for TTA), see curve characteristics in Fig. 2B and Table 3. TTA with MPF-M showed decreased knee flexion moments compared to ESR (peak moment p < 0.05, r = 0.70). Controls showed a partially different behavior using larger flexion moments at the first peak of the double hump.

**Table 3:** Sagittal joint moments for the prosthetic side (TTA, TFA) and controls

	**TFA**		**TTA**		**Controls**
	**ESR**	**MPF-M**	**ESR**	**MPF-M**	**-**
*Prosthetic side—UP*					
Sagittal ankle moment at SA = 0 (Nm/kg)	**0.92 ± 0.05**	**0.65 ± 0.18**	**0.83 ± 0.15**	**0.42 ± 0.16**	0.07 ± 0.05
Peak sagittal ankle moment (Nm/kg)	**1.45 ± 0.18**	**1.59 ± 0.15**	1.26 ± 0.18*	1.45 ± 0.18	1.69 ± 0.13
Sagittal knee moment at SA = 0 (Nm/kg)	**0.72 ± 0.16**	**0.53 ± 0.11**	**0.55 ± 0.15**	**0.28 ± 0.20**	-0.41 ± 0.21
Peak sagittal knee moment (Nm/kg)	0.74 ± 0.14	0.68 ± 0.12	**0.70 ± 0.17**	**0.45 ± 0.17**	0.65 ± 0.14
*Prosthetic side—DOWN*					
Sagittal ankle moment at SA = 0 (Nm/kg)	− 0.11 ± 0.11	− 0.18 ± 0.11*	− **0.20 ± 0.08**	**0.02 ± 0.22**	0.01 ± 0.13
Peak sagittal ankle moment mid-late stance (Nm/kg)	**1.27 ± 0.11**	**1.18 ± 0.07**	1.08 ± 0.19^÷^	1.17 ± 0.08^÷^	1.32 ± 0.14
Sagittal knee moment at SA = 0 (Nm/kg)	− 0.28 ± 0.16	− 0.34 ± 0.15	− 0.22 ± 0.20	− 0.13 ± 0.19*	− 0.42 ± 0.15
Peak sagittal knee moment (Nm/kg)	− 0.85 ± 0.14	− 0.78 ± 0.07	− **0.53 ± 0.27**	− **0.35 ± 0.28**^**÷**^	− 0.81 ± 0.15

Statistical differences between feet within a group (TTA, TFA) marked bold (p < 0.05). Values for controls are given for a qualitative comparison. SA = 0—vertical shank orientation. Ankle moments: positive—dorsiflexing, negative—plantar flexing; knee moments: positive—extending, negative—flexing

For Down, TTA and TFA utilized more upright shank orientations compared to Up at heel strike while controls did not change them to the same extent. For controls, almost the same ankle moments close to zero were found for Down and Up at vertical shank orientation (0.01 Nm/kg and 0.07 Nm/kg). Dorsiflexion moments similar to those of controls were found for TTA with MPF-M. In contrast, plantarflexion moments were observed in TTA (p < 0.01, r = 0.89 compared to MPF-M) and TFA with ESR as well as in TFA with MPF-M. For Down, the ankle moment pulled the shank in an upright position (controls, TTA with MPF-M) and beyond (TTA and TFA with ESR, TFA with MPF-M). All knee moments found at vertical shank position were flexion moments, see Table 3.

## Discussion

Ramp walking as part of independent community ambulation is a challenging situation for persons with lower-limb amputation. New prosthetic technologies and concepts have been designed to help reduce these challenges. In this study, the MPF-M adapted kinematics and kinetics for TTA and TFA to facilitate ramp walking. Most prominent improvements to ESR feet with rigid ankle attachments were an increased and situation-adapted ankle ROM, a dorsiflexed ankle during swing and reduced ankle and knee moments that may make it easier to walk up slopes and control downhill gait.

### MPF-M influenced kinematics and kinetics of ramp ambulation

Ankle-angle characteristics of prosthetic feet, influenced by carbon keel stiffness and design, have been identified as an important factor for ramp walking [5, 9, 15, 28, 29]. In this study, the ankle ROM was increased by approximately 35% for Up (TTA, TFA) and Down (TTA) with the MPF-M compared to ESR feet. A previous study with the same MPF had reported an even greater ankle ROM during slope walking compared to participants´ prescribed prosthetic feet [9].

Another major ankle characteristic of the MPF-M is that the foot remained in dorsiflexion after push-off for the entire swing phase in both slope conditions. This concurs with the findings of Schmalz et al. [9]. Such an ankle behavior is associated with an increased toe clearance and, therefore, likely with a reduced risk of tripping-related balance loss [30]. Similar strategies to increase toe clearance have been implemented in other hydraulic and/or microprocessor-controlled feet [13, 30, 31]. In particular, the dorsiflexed position during swing might also be helpful for ascending a ramp. In this study, persons without an amputation used a similarly dorsiflexed ankle position of about 5° or half of the slope angle at initial contact for Up. However, they also utilized a much more flexed knee joint and a more vertical shank orientation, which, in conjunction with the ankle angle, led to an almost immediate foot-flat after initial contact.

The initial knee flexion and the following stance-phase knee behavior revealed kinematic differences between all groups for ascending the slope that were independent of foot type. Similar adaptations in the walking pattern have been reported in other studies [21, 32, 33]. The participants with an amputation walked with an almost straight knee at heel strike and, in contrast to controls, only small (TTA) or no (TFA) stance knee flexion and, therefore, showed an inverted-pendulum walking behavior [34, 35] on the prosthetic side. Participants with TTA could use larger knee flexion angles but seemed to prefer more extended knees, maybe due to weakened leg muscles [36]. Furthermore, the control of the prosthesis under high loads and the force transmission to the socket might be easier with an extended knee.

The external sagittal joint moments on the prosthetic side that were most noticeably influenced by the foot type were observed at the knee of TTA. We found a significant reduction in peak moments of about 35% for TTA (Up, Down) compared to only about 8% for TFA (UP; not significant). However, that comparison does not reflect the differences between the curve characteristics at 10–40% GC that were even larger in parts and significant for TFA as well. Controls used a distinct flexion–extension knee moment characteristic for Up. In contrast, participants with an amputation showed almost exclusively extension moments until swing initiation. The reduction in the moment when using the MPF-M may reduce the strain to the residual knee joint of TTA and, thus, might help prevent knee overuse long-term. The decrease in the flexion moment that was observed for Down may contribute to a reduction in muscular effort of the residual limb to control the descent (TTA with MPF-M).

### Ankle control and the shank angle as a potential control parameter

Joint moments are typically reported as a function of time in the gait cycle. However, investigating knee and ankle moments with respect to the shank orientation may reveal additional information. Shultz and Goldfarb [17] used such an approach to demonstrate that the ankle moment as a function of the shank angle during 15–40% GC is likely an invariant in human walking over uneven ground. The results of the present study indicate that able-bodied individuals seem to adapt their locomotor system to achieve an ankle moment of about zero for a vertically orientated shank, independent of slope ascent or descent. In other words, the neutral point as a function of the ankle angle is adapted to the ground inclination while the neutral point as a function of the shank angle is rather constant.

ESR feet with rigid ankle attachments cannot adapt their neutral point and, thus, it occurs at an almost constant ankle angle independent of the degree of inclination [12]. Consequently, it shifts the ankle moment curves in negative (Up, dorsiflexion moments at SA = 0) or positive (Down, plantarflexion moments at SA = 0) direction at the vertical shank position, Fig. 3. The lack of adaptation makes it harder for Up to rotate the shank from its initial position forward to a vertical position. As the leg is almost extended at that point, the same can be assumed for the movement of the body´s center of mass. Contrary for Down, the plantarflexing moment pulls the shank forward into knee flexion. That is no problem for TFA with an appropriate knee joint (e.g. with advanced yielding function [37]) but very demanding for TTA. The knee flexion moments are controlled by the knee extensors which are usually weaker than in able-bodied individuals [36, 38].

The MPF-M used in this study adjusted its hydraulic plantar- and dorsiflexion resistances to the slope and, thus, changed its neutral point as a function of the ankle angle accordingly. In TTA during Down, it shifted the zero-crossing of the ankle moment curve towards a vertical shank orientation and, thus, mimicked the natural behavior. Since the shank angle is used as a control parameter it would be conceivable that this specific shift is a control goal. For Up, however, a similar shift is not visible. Nonetheless, the MPF-M control reduced the ankle moment, which facilitates the forward rotation of the shank over the foot. To realize a similar adaptation with a non-hydraulic ESR foot, it would have to be dorsiflexed for Up and plantarflexed for Down. It was shown that such adaptations can influence the prosthetic side knee moment in TTA positively when ascending a slope [13, 21].

### Different settings for individuals with TTA and TFA

Differences in the knee moments between feet were found to be negligible for Down in the TFA group but substantial in the TTA group. For descending, participants with TFA utilized the enhanced yielding mode of the MPK that enables continuous flexion of the knee and decelerates slope descent through hydraulic damping. For these subjects, the knee joint is likely the more important component with regard to safety and walking modes [24, 37]. Our results indicate that the MPF-M acted like a “regular” prosthetic foot for descending a slope, probably to not interfere with the MPK’s yielding mode. One of the few studies that investigated TFA slope walking with a MPF could not find benefits in ramp walking, although changes in the prosthetic ankle characteristics were reported [10]. In that study, participants with TFA showed neither knee stance flexion for Up nor continuous knee flexion for Down on a 5° slope that would be managed with normal knee characteristics by able-bodied controls. This further illustrates the importance of the prosthetic knee in facilitating walking of individuals with TFA. A coupling and coordinated control of both the MPF and MPK using, for instance, joint synergies and active push-off may be a step to further improve TFA gait on slopes and uneven ground.

### Limitations

The sample size of this study was relatively small with 7 individuals each with TTA and TFA, which limits the statistical power and generalizability of results. Especially TTA are known for larger inter-individual differences in their gait characteristics that may not have been fully represented in our sample. Another limitation of the study was the length of the slope. For Up, we captured only the first ground contact of the prosthetic foot with the slope. Subsequent steps might have shown different adaptations of the MPF-M in terms of shifting the zero-crossing of the ankle moment curve further towards its vertical shank position. Also, adaptations of the user to the slope could potentially change with later steps.

The effect of the MPF-M on the sound side was not investigated in this study. Future studies should investigate possible effects of the feet on the sound side for different amputation levels and situations.

## Conclusion

The MPF-M facilitated walking on slopes by adapting instantaneously to terrain inclinations and, thus, easing the forward rotation of the leg over the prosthetic foot compared to ESR feet with a fixed ankle attachment, possibly making it easier to walk up a slope and to control the gait speed when descending. It assumed a dorsiflexed position during swing and enabled a larger ankle ROM and reduced the moments acting on the residual knee, which might help reduce knee overuse long-term. For individuals with TFA, the prosthetic knee joint seems to play a more important role than the foot for walking on ramps.

## Data Availability

All data generated and analysed during this study are included in this published article (in form of figures and tables) or in the supplement.

## Abbreviations

GRF: Ground reaction force
TTA: (Persons) with a transtibial amputation
TFA: (Persons) with a transfemoral amputees
Controls: Persons without an amputation (reference group)
MPF: Microprocessor-controlled prosthetic feet
MPF-M: In this study used MPF
MPK: Microprocessor-controlled prosthetic knee
Up: Upward slope
Down: Downward slope
ROM: (Ankle) range of motion
SA: Shank angle
